# Secretory Acid Sphingomyelinase in Children and Adolescents With Type 1 Diabetes

**DOI:** 10.1155/jdr/5569377

**Published:** 2026-03-27

**Authors:** Chiara Mameli, Alice Bolchini, Cristina Ferrigno, Paulina Roux Biejat, Alessandro Arcari, Silvia Zecchini, Alessandra Napoli, Francesca Chiara Redaelli, Maddalena Macedoni, Agnese Petitti, Gianvincenzo Zuccotti, Emilio Clementi, Cristiana Perrotta

**Affiliations:** ^1^ Department of Pediatrics, Vittore Buzzi Children’s Hospital, ASST Fatebenefratelli Sacco, Milan, Italy, asst-fbf-sacco.it; ^2^ Department of Biomedical and Clinical Sciences, Università degli Studi di Milano, Milan, Italy, unimi.it; ^3^ E. Medea Scientific Institute, Bosisio Parini, Italy, emedea.it

**Keywords:** acid sphingomyelinase, children, established T1D, new-onset T1D, T1D, type 1 diabetes

## Abstract

**Introduction:**

The activity of acid sphingomyelinase (ASMase), a key enzyme in sphingolipid metabolism, has been found to be increased in a variety of human diseases. Studies conducted on animal and cellular models showed that sphingolipids and ASMase play a central role in the pathogenesis of type 1 diabetes (T1D) and T1D‐related vascular damage. Currently, no studies have investigated the role of ASMase activity in pediatric patients with T1D. Therefore, we conducted a cross‐sectional study to evaluate the activity of the secretory form of ASMase (S‐ASMase) in the serum of patients with T1D aged 2–16 years in comparison with a control group (healthy subjects matched for age, gender, and pubertal stage).

**Materials and Methods:**

We recruited children and adolescents affected by T1D (including patients with new‐onset and established T1D) aged 2–16 years and healthy normal‐weight subjects with normal timing of puberty (matched for age, gender, and pubertal stage), who were consecutively admitted—as outpatients—to our institution for screening purposes. Serum lipid profile, glycated hemoglobin (HbA1c), and urine albumin–creatinine ratio (uACR) were assessed in all T1D patients. S‐ASMase activity was measured in all study participants through a colorimetric assay.

**Results:**

In total, 68 T1D patients and 51 healthy controls were recruited in this study. None of the T1D patients had T1D‐related complications. No difference in S‐ASMase activity was observed between subjects with T1D and healthy controls. However, when T1D patients were stratified according to the duration of diabetes, we found a significantly higher activity of S‐ASMase in patients with new‐onset T1D (recruited within 1 week after the disease diagnosis) as compared to that observed in patients with established T1D. In all patients with T1D, S‐ASMase activity correlated positively with HbA1c and triglyceride levels, while it correlated negatively with total cholesterol (TC) and high‐density lipoprotein cholesterol (HDL‐C) levels. However, there were no significant differences in S‐ASMase activity between T1D patients with new‐onset disease who presented with diabetic ketoacidosis (DKA; *n* = 12) and T1D patients with new‐onset disease who did not present with DKA (*n* = 13).

**Conclusion:**

Our study evaluated, for the first time, the in vivo activity of S‐ASMase in a pediatric cohort of patients with T1D. In pediatric patients with new‐onset T1D, we found a significantly higher S‐ASMase activity as compared to that observed in patients with established T1D. In all T1D patients, the positive correlation between S‐ASMase activity, HbA1c, and triglyceride levels, as well as the negative correlation between S‐ASMase activity and HDL‐C levels, suggests a potential role played by sphingolipids in T1D pathophysiology. Further mechanistic studies are needed to better elucidate the role of S‐ASMase in patients with T1D at different stages of the disease.

## 1. Introduction

Sphingolipids represent a class of lipids that serve as structural molecules of cell membranes and can also act as bioactive signaling molecules [1]. Acid sphingomyelinase (ASMase) is a key enzyme in sphingolipid metabolism, catalyzing the hydrolysis of cell membrane sphingomyelin to ceramide and phosphorylcholine [2, 3]. The activity of ASMase in blood—mainly represented by the secretory form of ASMase (S‐ASMase) in the serum—is increased in pathological conditions characterized by a marked inflammatory activity as a response to cell stress [4]. Studies conducted on cellular and animal models highlighted the central role of various types of sphingolipids in the pathogenesis of type 1 diabetes (T1D) [5–7]. In particular, ceramide appears to exert diabetogenic effects through three main mechanisms, namely, induction of beta‐cell apoptosis, increase in insulin resistance, and reduction of insulin gene expression [8]. Ceramide is also implicated in the development of microvascular complications of diabetes mellitus through the upregulation of inflammatory cytokines and induction of endothelial dysfunction [9–12].

It has been shown that ASMase expression level is significantly increased in human retinal endothelial cells and CD34+ circulating angiogenic cells and plasma of patients with type 2 diabetes (T2D) as compared to control donor tissue [11]. Moreover, it has been found that vascular ASM is increased in the retinas of animal models during the vasodegenerative phase of diabetic retinopathy [12]. ASMase has also been shown to promote diabetic cardiomyopathy via dysregulation of mitochondrial calcium homeostasis in mouse models of T2D [13]. To date, no studies have investigated the role of S‐ASMase activity in pediatric patients with T1D. Therefore, the main aim of the present study was to evaluate the activity of S‐ASMase in a cohort of children and adolescents affected by T1D and in healthy controls. Moreover, we investigated the correlation between the activity of S‐ASMase and different clinical and laboratory parameters assessed in our cohort of T1D patients.

## 2. Materials and Methods

### 2.1. Study Design and Participants

From January 2023 to December 2023, this cross‐sectional study enrolled T1D patients who were consecutively admitted to the Diabetes Clinic of the Vittore Buzzi Children’s Hospital, including patients with new‐onset T1D. The inclusion criteria were the following: diagnosis of T1D according to the International Society for Pediatric and Adolescent Diabetes (ISPAD) Clinical Practice Consensus Guidelines [14]; intensive insulin therapy; age between 2 and 16 years. Patients with new‐onset T1D were recruited within 1 week after the disease diagnosis, while T1D patients with established disease were defined as those diagnosed with the disease more than 6 months prior to enrollment. The exclusion criteria were the following: T2D; neonatal diabetes; maturity‐onset diabetes of the young (MODY); syndromic forms of diabetes; smoking. Moreover, we excluded patients with ASMase deficiency and coexisting diseases. Moreover, the study included a control group (ctr) of healthy normal‐weight subjects with normal timing of puberty and without diabetes and other chronic diseases (subjects matched for age, gender, and pubertal stage), who were consecutively admitted—as outpatients—to the Vittore Buzzi Children’s Hospital (for screening purposes).

The study was approved by the local ethics committee (Approval Code: 321/2021) and conducted according to the Declaration of Helsinki. Written informed consent to participate in the study was obtained from the parents of the study subjects. Assent to participate in the study was also obtained from the study subjects.

### 2.2. Clinical Assessment

For each T1D patient, data regarding the following demographics and parameters were collected at the time of recruitment: gender, age, height, body weight, body mass index (BMI), age at the time of T1D diagnosis, duration of diabetes, and presence of diabetic ketoacidosis (DKA) at the disease onset. For each participant in the control group, data regarding the following demographics and parameters were collected at the time of recruitment: gender, age, height, body weight, and BMI. Participants’ body weight was measured using a digital scale (SECA, Hamburg, Germany), while the participants’ height was measured using a Harpenden stadiometer (SECA, Hamburg, Germany).

BMI was calculated by dividing the body weight in kilograms by the height in meters squared (kg/m^2^). The standard deviation scores (SDSs) for BMI (BMI *Z*‐score) were calculated using the World Health Organization (WHO) charts (WHO growth standards for children aged 0–5 years and WHO growth reference for those aged 5–19 years) [15, 16]. For children under 5 years of age, overweight and obesity were defined using the WHO weight‐for‐age and weight‐for‐height cut‐off values, as follows: underweight was defined as weight‐for‐age lower than 2 standard deviations (SDs) below the WHO Child Growth Standards median; overweight was defined as weight‐for‐height greater than 2 SDs above the WHO Child Growth Standards median; obesity was defined as weight‐for‐height greater than 3 SDs above the WHO Child Growth Standards median [17]. For children aged 5–19 years, weight status categories were defined according to WHO BMI‐for‐age cut‐off values, as follows: underweight was defined as a BMI *Z*‐score lower than 2 SDs below the WHO growth reference median; overweight was defined as BMI‐for‐age greater than 1 SD above the WHO growth reference median; obesity was defined as BMI‐for‐age greater than 2 SDs above the WHO growth reference median [17]. BMI *Z*‐scores were used as continuous variables in all correlation analyses. Systolic blood pressure (SBP) and diastolic blood pressure (DBP) were measured following international guidelines in all study participants, and hypertension was defined as SBP and/or DBP (on repeated measurements) at or above the 95th percentile for age, sex, and height [18].

### 2.3. Blood and Urine Tests

Blood samples were collected after a 10‐h overnight fast, and sera were obtained for the measurement of the following parameters: glycated hemoglobin (HbA1c), total cholesterol (TC), high‐density lipoprotein cholesterol (HDL‐C), low‐density lipoprotein cholesterol (LDL‐C), triglycerides, and activity of S‐ASMase. Serum lipid profile and HbA1c were assessed only in T1D patients. HbA1c was measured using a fully automated high‐performance liquid chromatography system (Variant II, Bio‐Rad Laboratories, Munich, Germany). A first morning urine sample was obtained for all T1D patients in order to measure the urine albumin–creatinine ratio (uACR) and to evaluate the presence of albuminuria. All blood and urine samples were analyzed by the laboratory of the ASST Fatebenefratelli Sacco (Via G.B. Grassi 74, Milan, Italy).

#### 2.3.1. Assessment of S‐ASMase Activity in the Blood Serum

The activity of S‐ASMase in the serum of T1D patients and healthy controls was assessed through a colorimetric assay, measuring the conversion of sphingomyelin to phosphorylcholine according to the manufacturer’s instructions (BioVision, Inc.; Acid Sphingomyelinase Activity Colorimetric Assay Kit; Milpitas, CA, USA), with some modifications. Briefly, blood was centrifuged (for 10 min, at 2000 × *g* and at room temperature), and serum was stored at −80°C for the following S‐ASMase activity test. S‐ASMase activity for each sample was assayed in 96‐well plates using an acid sample buffer (pH 5) containing Zn^2+^ (500 µM ZnCl_2_). Absorbance was measured in duplicate through a spectrophotometer (OD 570 nm). The S‐ASMase enzyme activity (expressed in pmol/mL/h) was calculated using a standard curve generated by measuring the absorbance of known choline concentrations. The amount of choline produced in each sample was determined from the absorbance values by interpolating the corresponding *x*‐axis value from the standard curve [19].

### 2.4. Ophthalmic Examination

All T1D patients underwent a complete ophthalmic examination (including slit lamp examination and fundoscopic examination) performed by an expert ophthalmologist to exclude the presence of diabetic retinopathy.

### 2.5. Statistical Analysis

Descriptive statistics (mean, SD, median, interquartile range [IQR], counts, and percentages) were used for the description of the study population. Deviation from normal data distribution was assessed using the D’Agostino–Pearson normality test. Results were reported as mean ± SD when distributions were approximately symmetric/normal and as the median and IQR when distributions were nonnormal. Inferential statistical analysis used to assess the statistical significance of differences between the study groups included the following tests: analysis of variance (ANOVA) or Kruskal–Wallis test followed by the Dunn’s post hoc test for multiple comparisons, Student’s *t*‐test or Welch’s *t* test or Mann–Whitney test for single comparisons, Pearson or Spearman correlations (all two‐tailed), as appropriate, with a *p*‐value less than 0.05 being considered statistically significant. Given the small sample size, the Fisher’s exact test was applied to ensure that the recruited participants were evenly distributed across relevant groups (e.g., sex and patient/control status). The Grubbs’s test was used to identify possible outliers. The GraphPad Prism software Version 10.2.0 (GraphPad Software Inc., San Diego, CA, USA) was used to perform the statistical analysis.

## 3. Results and Discussion

We recruited 68 T1D patients (mean age: 10.19 ± 3.99 years; 31 [45.6%] males) and 51 healthy controls (mean age: 10.78 ± 3.46 years; 25 [49%] males). Of the 68 T1D patients, 43 had established disease and 25 had new‐onset disease. Of the 25 patients with new‐onset T1D, 12 (48%) presented with DKA. Table 1 shows the baseline characteristics of the study participants. None of the T1D patients were affected by underweight, while 12 (17.64%) and 4 (5.88%) T1D patients were affected by overweight and obesity, respectively. None of the T1D patients had diabetes‐related complications, including diabetic retinopathy, microalbuminuria, and hypertension. All T1D patients and healthy controls were nonsmokers. There was no significant difference in the mean age between T1D patients and healthy controls. Moreover, there was no significant difference in the percentage of males and females between T1D patients and healthy controls (*p* = 0.7154; Fisher’s exact test).

**Table 1:** Baseline characteristics of the study participants.

Characteristics	T1D patients (*n* = 68; 43 patients with established disease; 25 patients with new‐onset disease)	Healthy controls (*n* = 51)	*p*‐Value
Age (years)			
Mean ± SD	10.19 ± 3.99	10.78 ± 3.46	0.3944
Gender			
Male, *n* (%)	31 (45.6%)	25 (49%)	0.7154
Weight (kg)			
Median (IQR)	42 (27.7)	31.2 (21.6)	0.0706
Height (cm)			
Median (IQR)	148 (28)	136.5 (30.1)	0.0309
BMI *Z*‐score			
Mean ± SD	0.398 ± 0.880	−0.061 ± 0.898	0.0061
HbA1c (%)			
Median (IQR)	7.70 (3.9)	N/A	N/A
Triglycerides (mg/dL)			
Median (IQR)	60 (31)	N/A	N/A
TC (mg/dL)			
Mean ± SD	163.5 ± 29.6	N/A	N/A
HDL‐C (mg/dL)			
Mean ± SD	50.85 ± 13.93	N/A	N/A
LDL‐C (mg/dL)			
Mean ± SD	98.95 ± 26.13	N/A	N/A

*Note:* Results are presented as median (IQR) or mean ± SD or number (percentage). HbA1c, glycated hemoglobin.

Abbreviations: BMI, body mass index; HDL‐C, high‐density lipoprotein cholesterol; IQR, interquartile range; LDL‐C, low‐density lipoprotein cholesterol; *n*, number; N/A, not available; SD, standard deviation; TC, total cholesterol; T1D, type 1 diabetes.

**Figure 1:**
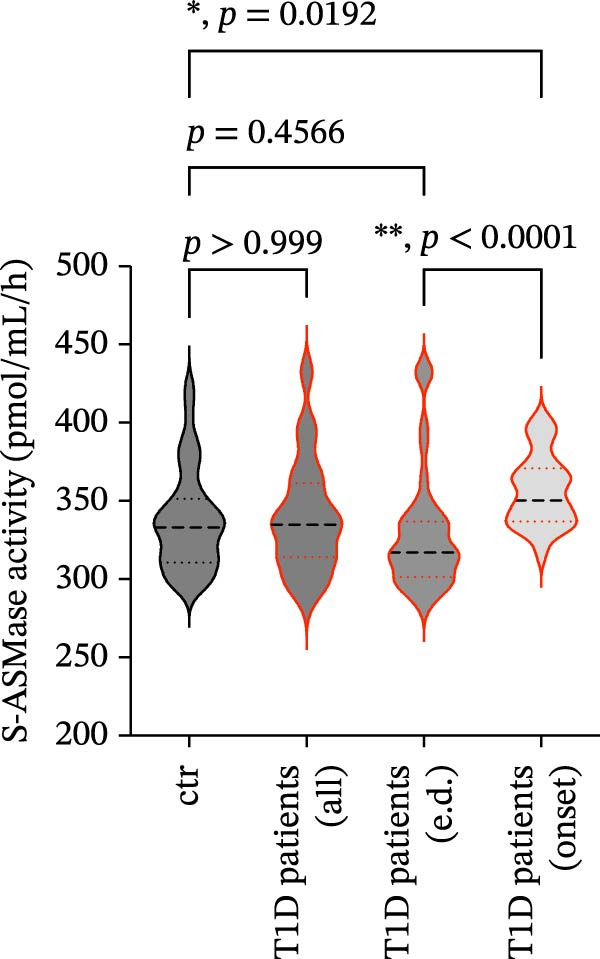
Distribution of S‐ASMase activity in healthy controls and T1D patients. Healthy controls (ctr; *n* = 51); all T1D patients enrolled (all; *n* = 68); T1D patients with established disease (e.d.; *n* = 43); T1D patients with new‐onset T1D (onset; *n* = 25).  ^∗^ *p*  < 0.05;  ^∗∗^ *p*  < 0.0001; statistical analysis was performed using the Kruskal–Wallis test followed by the Dunn’s post hoc test for multiple comparisons. S‐ASMase, secretory form of acid sphingomyelinase; T1D, type 1 diabetes.

The activity of S‐ASMase did not differ significantly between T1D patients and healthy controls (Figure 1). However, when we stratified T1D patients according to the time of disease onset (patients with established disease and patients with new‐onset disease), we found a significantly higher S‐ASMase activity in patients with new‐onset disease as compared to those with established disease and to healthy controls; conversely, the latter two groups did not differ in terms of S‐ASMase activity (Figure 1). This pattern of S‐ASMase activity may reflect compensatory adaptations that emerge during the chronic progression of the disease, counterbalancing the early increase observed at diagnosis.

Since the early stage of the disease, T1D is characterized by high circulating levels of pro‐inflammatory cytokines and mediators such as interleukins (IL‐1, IL‐6, and IL‐12), interferons (such as IFN‐α and IFN‐γ), tumor necrosis factor alpha (TNF‐α), and nitric oxide (NO) [20–27]. In T1D, TNF‐α, IL‐1β, and IFN‐γ exert cytotoxic effects on pancreatic beta cells, ultimately inducing beta‐cell apoptosis and therefore contributing to the pathogenesis of the disease [28, 29]. Evidence from in vitro studies suggests that ceramide, synthesized de novo or generated through the sphingomyelinase‐dependent hydrolysis of sphingomyelin, plays a role in beta‐cell apoptosis, including beta‐cell apoptosis induced by pro‐inflammatory cytokines [8, 30–32]. Moreover, ceramide is known to play a role in inflammatory processes by triggering and regulating immunity [33]. Indeed, ceramide signaling in immune cells critically regulates cell activation, differentiation, survival, and death, for example, by inducing macrophage polarization toward a pro‐inflammatory M1 phenotype and stimulating dendritic cell activation [34, 35]. Moreover, elevated ceramide levels have been associated with the activation of pro‐inflammatory signaling cascades, such as the nuclear factor kappa B (NF‐κB) and NLRP3 inflammasome pathways [36–39]. Most of the pro‐inflammatory activities of ceramide are mediated by its direct metabolite ceramide‐1‐phosphate and by sphingosine‐1‐phosphate (S1P), which is generated through ceramidase‐mediated degradation of ceramide to form sphingosine and subsequent sphingosine kinase‐mediated phosphorylation of sphingosine [40, 41]. Interestingly, some studies conducted in mice suggested that the S1P receptor 1 pathway may be involved in the pathogenesis of T1D [5, 6].

We did not find a statistically significant difference in S‐ASMase activity between males and females of the entire T1D group (Figure 2A). Moreover, we did not observe any significant correlation of S‐ASMase activity with age (correlation coefficient [*r*] = −0.04337, *p* = 0.7255) and BMI *Z*‐score (*r* = 0.07702, *p* = 0.5452) in the entire T1D group (Figure 2B and C). The absence of correlation of S‐ASMase activity with the BMI *Z*‐score appears to be in contrast with our previous study, where we found a significantly higher S‐ASMase activity in pediatric patients with obesity as compared to healthy pediatric controls [19]. However, according to the mean (±SD) values of the BMI *Z*‐score (0.398 ± 0.880), T1D patients of the present study can mostly be classified as normal weight, with only 5.88% of the T1D subjects recruited in the study being affected by obesity according to the WHO charts [15, 16].

Figure 2Comparison of S‐ASMase activity between female and male T1D patients, and correlation of S‐ASMase activity with age and BMI *Z*‐score among T1D patients. (A) S‐ASMase activity levels in female (*n* = 37) and male (*n* = 31) T1D patients. (B) Correlation between S‐ASMase activity and age in T1D patients. (C) Correlation between S‐ASMase activity and BMI *Z*‐score in T1D patients. The solid line represents the linear regression, while the dashed lines represent the 95% confidence interval (CI). BMI, body mass index; S‐ASMase, secretory form of acid sphingomyelinase.

**Figure:**
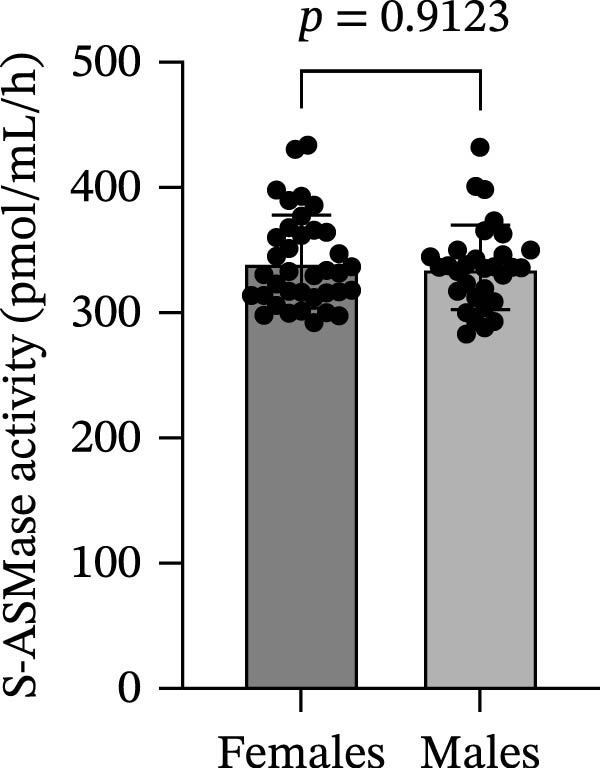
(A)

**Figure:**
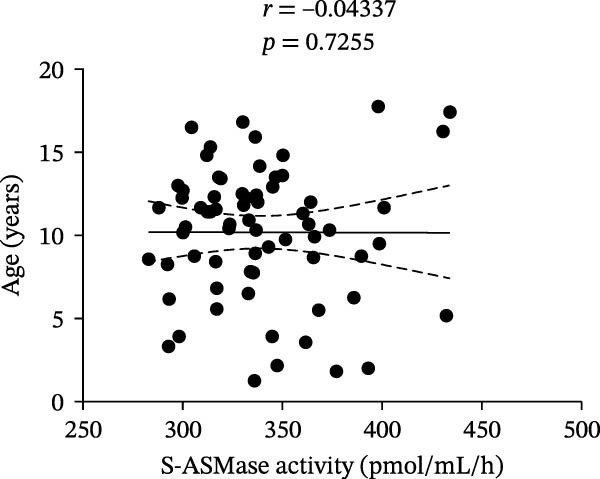
(B)

**Figure:**
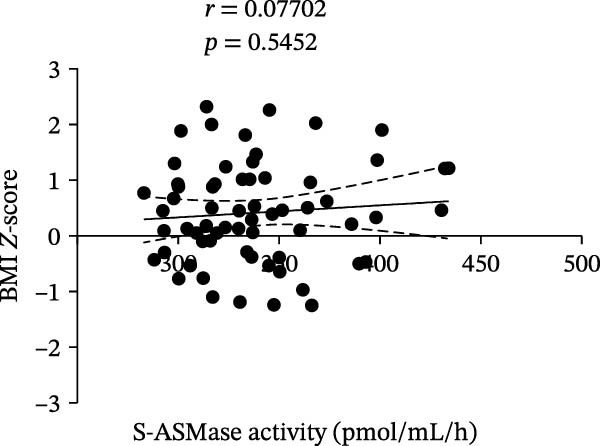
(C)

Among T1D patients, we found a positive correlation of S‐ASMase activity with HbA1c (*r* = 0.3934, *p* = 0.0010) and triglycerides (*r* = 0.4266, *p* = 0.0004) (Figure 3A and B), as well as a negative correlation of S‐ASMase activity with TC (*r* = −0.3137, *p* = 0.0103) and HDL‐C levels (*r* = −0.4471, *p* = 0.0002) (Figure 3C and D). S‐ASMase activity was not significantly correlated with LDL‐C levels (*r* = −0.2289, *p* = 0.0645).

Figure 3Correlation of S‐ASMase activity with HbA1c, triglycerides, TC, and HDL‐C in T1D patients. (A) Correlation between S‐ASMase activity and HbA1c in T1D patients. (B) Correlation between S‐ASMase activity and triglycerides in T1D patients. (C) Correlation between S‐ASMase activity and TC in T1D patients. (D) Correlation between S‐ASMase activity and HDL‐C in T1D patients. The solid line represents the linear regression, while the dashed lines represent the 95% confidence interval (CI).  ^∗^ *p*  < 0.05;  ^∗∗^ *p*  < 0.01;  ^∗∗∗^ *p*  < 0.001. HbA1c, glycated hemoglobin; HDL‐C, high‐density lipoprotein cholesterol; S‐ASMase, secretory form of acid sphingomyelinase; TC, total cholesterol.

**Figure:**
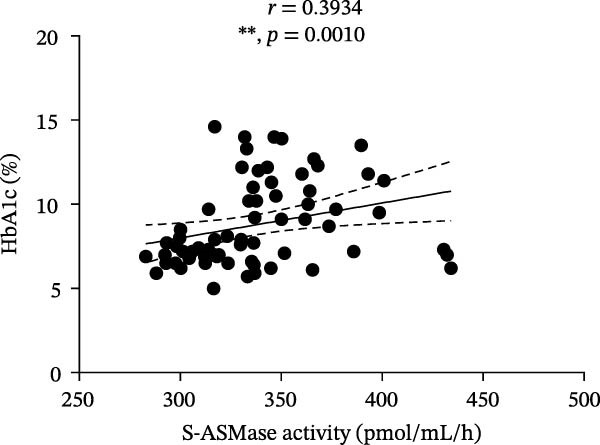
(A)

**Figure:**
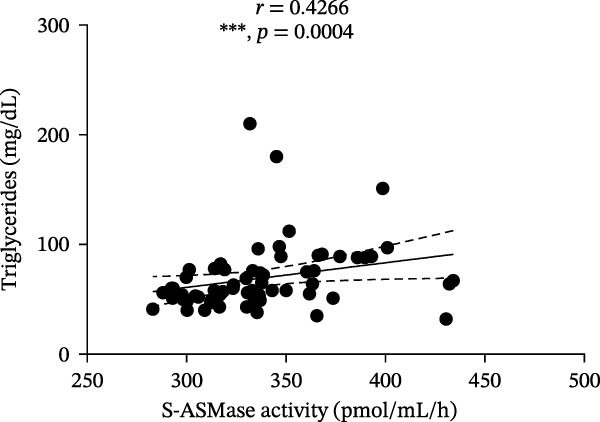
(B)

**Figure:**
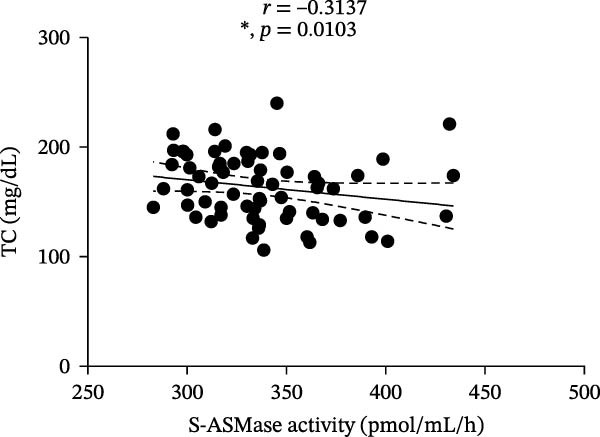
(C)

**Figure:**
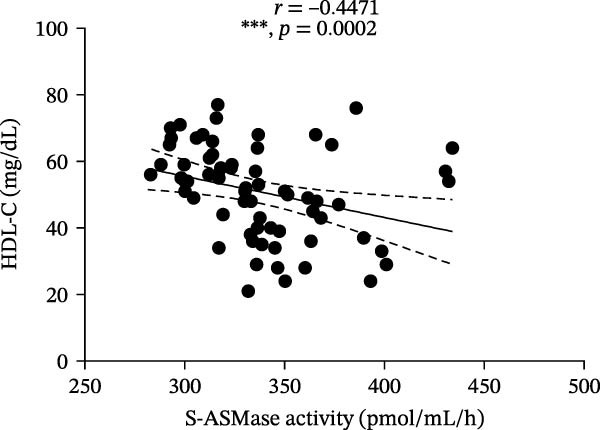
(D)

HbA1c is a well‐established biomarker used for the diagnosis and follow‐up of diabetes mellitus [42, 43]. Indeed, the main goal of insulin therapy in T1D is to avoid chronic hyperglycemia and prevent its long‐term adverse effects [44]. In this regard, the ISPAD guidelines recommend maintaining HbA1c levels below 53 mmol/mol (below 7.0%) for young people with diabetes in order to prevent microvascular and macrovascular complications of diabetes [45]. To the best of our knowledge, this is the first report regarding the existence of a correlation between S‐ASMase activity and HbA1c levels in patients with new‐onset and established T1D. Interestingly, vascular ASMase expression has been found to be increased in the retinas of rodent models of diabetic retinopathy during the vasodegenerative phase of the disease [12]. Moreover, Dorweiler et al. [46, 47] showed that anti‐ceramide immunotherapy may prevent pro‐inflammatory and proapoptotic changes in the retinal vasculature of rodent models of diabetic retinopathy, which are highly prevalent during the early stages of retinal vasculopathy .

We also observed significant differences in metabolic parameters between patients with newly diagnosed—not yet controlled—T1D and those with established T1D (Figure 4). As expected, median HbA1c values and triglycerides were significantly higher in T1D patients with new‐onset disease as compared to those with established disease: median HbA1c, 7.00% (IQR: 1.125) vs. 11.80% (IQR: 2.9) (*p*  < 0.0001); median triglycerides, 54.00 mg/dL (IQR: 21.5) vs. 82.00 mg/dL (IQR: 35.5) (*p* < 0.0001) (Figure 4A and B). Moreover, mean TC and HDL‐C values (±SD) were significantly lower in T1D patients with new‐onset disease as compared to those with established disease: mean TC, 171.6 ± 26.73 vs. 149.2 ± 29.02 mg/dL (*p* = 0.0028); mean HDL‐C, 58.59 ± 9.391 vs. 38.169 ± 10.41 mg/dL (*p*  < 0.0001) (Figure 4C and D). There were no statistically significant differences in mean LDL‐C values between T1D patients with new‐onset disease and those with established disease: mean LDL‐C, 94.92 ± 28.59 vs. 101.4 ± 24.55 mg/dL (*p* = 0.3508) (Figure 4E).

Figure 4Comparison of laboratory parameters between T1D patients with established disease and T1D patients with new‐onset disease, and comparison of S‐ASMase activity between patients with new‐onset T1D who presented with DKA and patients with new‐onset T1D who did not present with DKA. (A) Median HbA1c levels in T1D patients with established disease (e.d.; *n* = 43) and in T1D patients with new‐onset disease (onset; *n* = 25). (B) Median triglyceride levels in T1D patients with established disease (e.d.; *n* = 43) and and in T1D patients with new‐onset disease (onset; *n* = 25). (C) Mean TC levels in T1D patients with established disease (e.d.; *n* = 43) and in T1D patients with new‐onset disease (onset; *n* = 25). (D) Mean HDL‐C levels in T1D patients with established disease (e.d.; *n* = 43) and in T1D patients with new‐onset disease (onset; *n* = 25). (E) Mean LDL‐C levels in T1D patients with established disease (e.d.; *n* = 43) and in T1D patients with new‐onset disease (onset; *n* = 25). For the analysis of HbA1c and triglycerides data, which were not normally distributed, we used the Mann–Whitney test; for the analysis of TC, HDL‐C, and LDL‐C data, which were normally distributed, we used the Welch’s *t* test.  ^∗^ *p*  < 0.01;  ^∗∗^ *p*  < 0.0001. (F) S‐ASMase activity in patients with‐new‐onset T1D (onset) who presented with DKA (*n* = 12) and in those who did not present with DKA (*n* = 13). DKA, diabetic ketoacidosis; HbA1c, glycated hemoglobin; HDL‐C, high‐density lipoprotein cholesterol; LDL‐C, low‐density lipoprotein cholesterol; S‐ASMase, secretory form of acid sphingomyelinase; TC, total cholesterol; T1D, type 1 diabetes.

**Figure:**
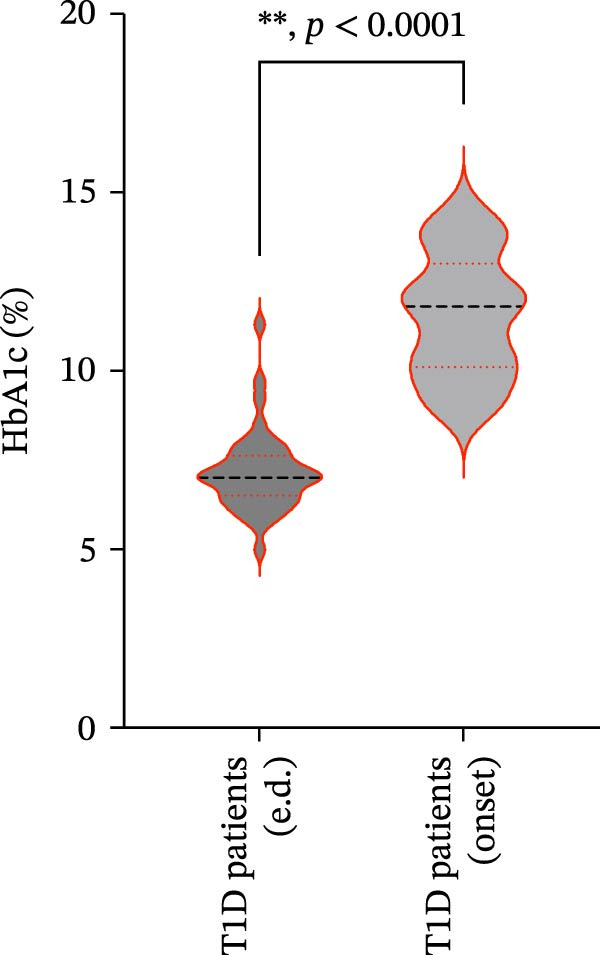
(A)

**Figure:**
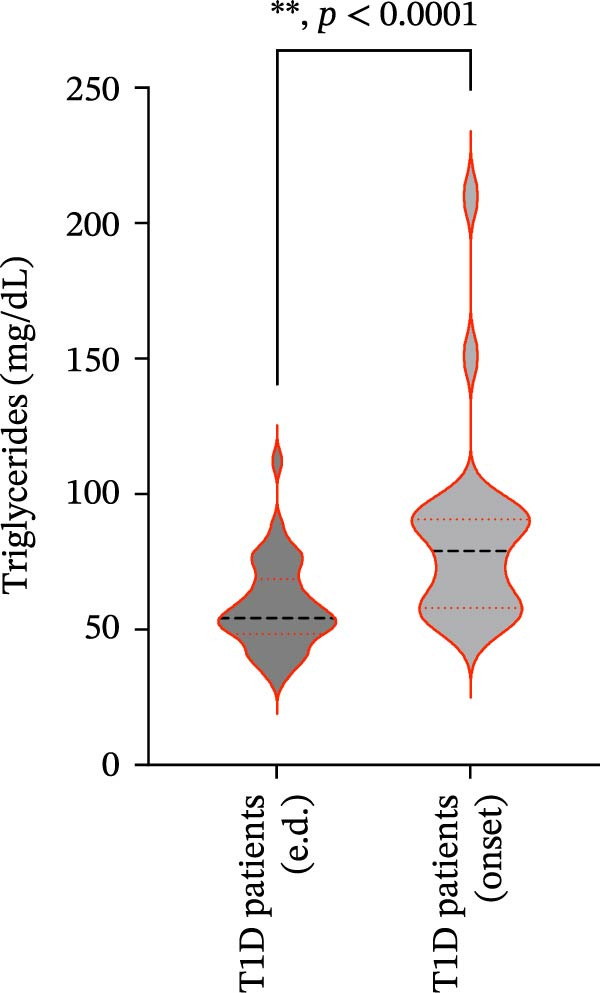
(B)

**Figure:**
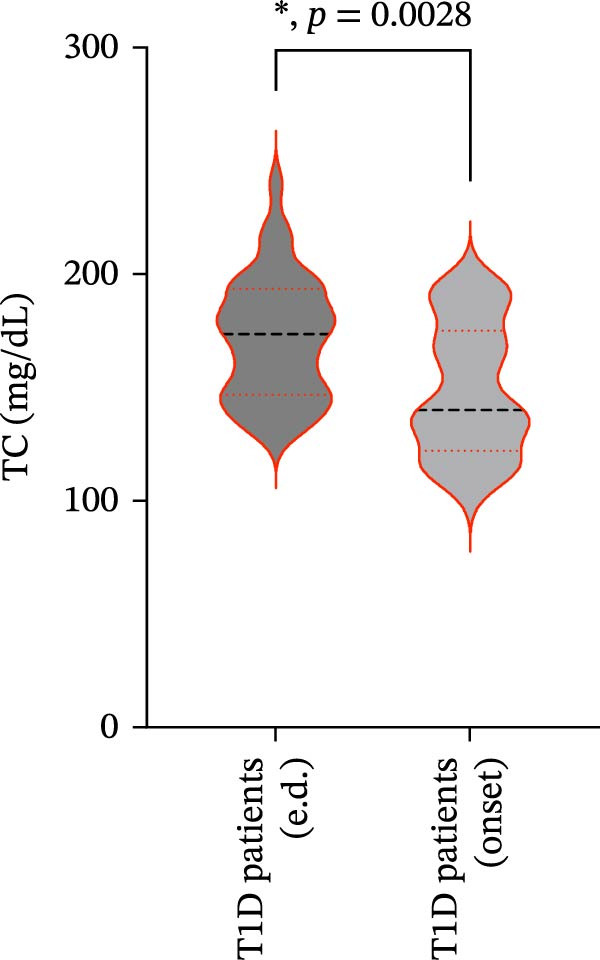
(C)

**Figure:**
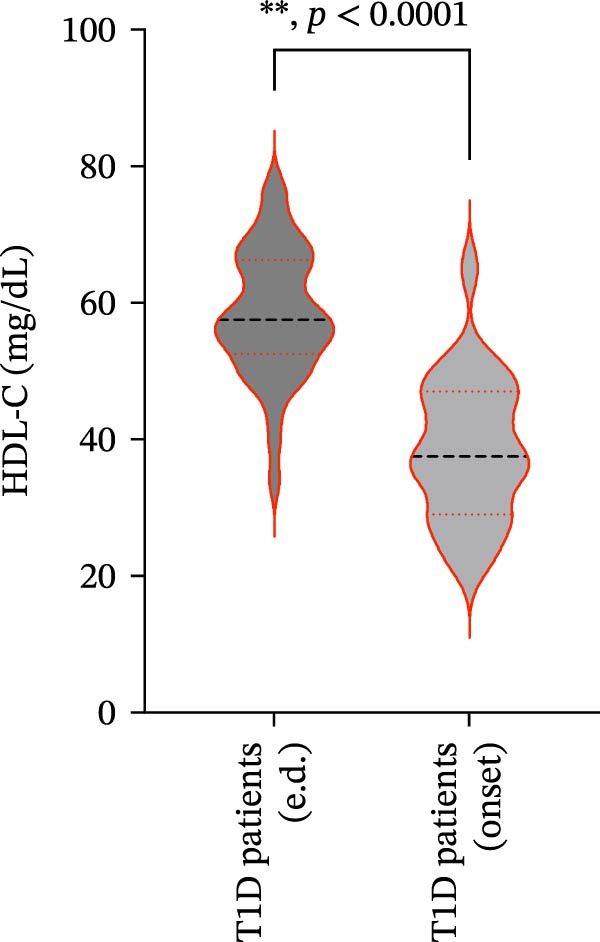
(D)

**Figure:**
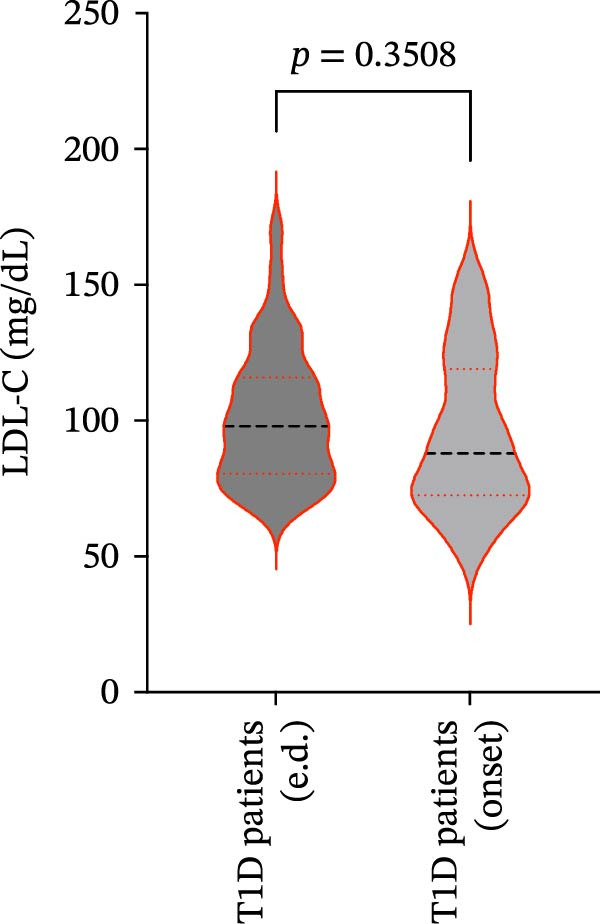
(E)

**Figure:**
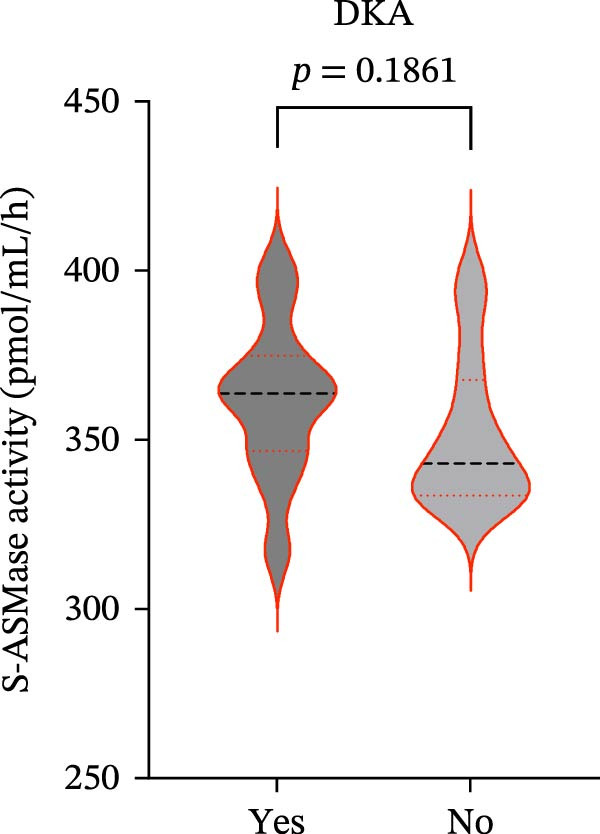
(F)

The association between T1D and circulating lipids still needs to be fully understood, considering that serum lipid alterations represent an additional risk factor for cardiovascular disease [48–50]. Abnormalities of circulating lipoproteins have been reported in T1D patients, particularly in the presence of poor glycemic control [51]. Of note, serum lipid alterations have been observed in T1D patients presenting with DKA, a life‐threatening condition resulting from insulin deficiency and often associated with hypertriglyceridemia [52, 53]. Recently, it has been demonstrated that achievement of optimal glycemic control after T1D onset leads to a significant reduction in serum concentrations of triglycerides and apolipoprotein (Apo) B‐containing lipoproteins [54]. Therefore, we also compared S‐ASMase activity between T1D patients with new‐onset disease who presented with DKA and T1D patients with new‐onset disease who did not present with DKA. The analysis revealed no significant differences in the S‐ASMase activity between T1D patients with new‐onset disease who presented with DKA (*n* = 12) and T1D patients with new‐onset disease who did not present with DKA (*n* = 13) (Figure 4F). However, the latter findings need to be confirmed on a larger sample of T1D patients.

Interestingly, a large study carried out in adult T1D patients has recently reported a strong correlation between the levels of different types of ceramides with HbA1c and triglycerides, indicating specific ceramides levels as potential biomarkers of future cardiovascular events and all‐cause mortality in individuals with long‐standing T1D [55].

We acknowledge that our study has some limitations, such as the relatively small sample size of the study population, the cross‐sectional nature of the study (which precludes the establishment of causality), as well as the lack of assessment of serum parameters in healthy controls, which prevented us from comparing the serum lipid profile between cases and controls.

## 4. Conclusions

In the last few years, sphingolipids and S‐ASMase have emerged as new risk factors in the development of T1D and its complications. However, the current paucity of studies prevents a comprehensive understanding of the pathophysiological role of the alterations in tissue and circulating sphingolipids levels in patients with new‐onset and established T1D. Our study investigated, for the first time, the activity of S‐ASMase in a cohort of pediatric patients with T1D, suggesting that alterations of sphingolipid metabolic pathways as indicated by higher S‐ASMase activity may already be present at the onset of the disease. Moreover, among T1D patients, the positive correlation between S‐ASMase activity, HbA1c, and triglyceride levels, as well as the negative correlation between S‐ASMase activity and HDL‐C levels, suggests a potential role played by sphingolipid in T1D pathophysiology. Thus, future mechanistic studies are needed to better elucidate the role of S‐ASMase in patients with T1D at different stages of the disease. Finally, the aforementioned observations pave the way for future studies aimed to explore whether alterations of sphingolipid metabolic pathways are also related to the development of T1D‐related chronic complications.

## Author Contributions

**Chiara Mameli**: writing – original draft, conceptualization, supervision, resources, funding acquisition, project administration. **Alice Bolchini and Cristina Ferrigno**: investigation, resources. **Paulina Roux Biejat and Alessandra Napoli**: investigation, methodology. **Alessandro Arcari**: investigation, formal analysis. **Silvia Zecchini**: investigation, visualization. **Maddalena Macedoni, Agnese Petitti, and Francesca Chiara Redaelli**: resources. **Gianvincenzo Zuccotti and Emilio Clementi**: writing – original draft, conceptualization, resources. **Cristiana Perrotta**: writing – original draft, conceptualization, supervision, resources, validation, funding acquisition, project administration.

## Funding

This study was supported by a grant from the University of Milan (Grant LINEA 2‐2019). Open access publishing facilitated by Universita degli Studi di Milano, as part of the Wiley ‐ CRUI‐CARE agreement.

## Conflicts of Interest

The authors declare no conflicts of interest.

## Data Availability

Data are available from the authors upon request.
